# Community Health Status Indicators: Adding a Geospatial Component

**Published:** 2008-06-15

**Authors:** Janet L Heitgerd, Andrew L Dent, Kimberlee A Elmore, Brian Kaplan, James B Holt, Marilyn M Metzler, Koren Melfi, Jennifer M Stanley, Keisher Highsmith, Norma Kanarek, Karen Frederickson Comer

**Affiliations:** Agency for Toxic Substances and Disease Registry; Agency for Toxic Substances and Disease Registry, Atlanta, Georgia; Agency for Toxic Substances and Disease Registry, Atlanta, Georgia; Agency for Toxic Substances and Disease Registry, Atlanta, Georgia; Centers for Disease Control and Prevention, Atlanta, Georgia; Centers for Disease Control and Prevention, Atlanta, Georgia; Association of State and Territorial Health Officials, Washington, District of Columbia; Public Health Foundation, Washington, District of Columbia; Health Resources and Services Administration, Rockville, Maryland; Johns Hopkins Bloomberg School of Public Health, Baltimore, Maryland; The Polis Center-IUPUI, Indianapolis, Indiana

## Abstract

An Internet mapping application is being introduced in conjunction with the release of the second version of the Community Health Status Indicators (CHSI) Report. The CHSI Geographic Information Systems (GIS) Analyst is an easy-to-use Web-based mapping application that provides new opportunities for the visualization, exploration, and understanding of the indicators. Indicators can be mapped and compared visually to other areas, including peer counties and neighboring counties. The Web site is accessible from a link on the CHSI Report Web site or directly from an Internet Web browser. In this paper, we discuss the conceptualization and implementation of this public health mapping application.

## Introduction

In July 2000, the first version of the Community Health Status Indicators (CHSI) Report was available on the Health Resources and Services Administration (HRSA) Web site. At that time, the display of geospatial data, while certainly not new, was not routinely a part of national public health projects and reports. When done at all, mapping was often thought of as a graphic to accompany the data and not as an enhancement of the data. Some notable exceptions to this are the National Center for Health Statistics' *Atlas of United States Mortality* (1) and the National Cancer Institute's *Atlas of Cancer Mortality in the United States, 1950–1994* (2). Much has changed since 2000, primarily because of the increased availability, affordability, and ease of use of geographic information systems (GIS) technology including software, hardware, and data for creating maps and hosting Web sites with a geospatial component. This has led to a proliferation of mapping Web sites that vary in intent, quality, and complexity, but nevertheless have helped to familiarize the public with the concept and purpose of mapping spatial relationships. Reference mapping sites emphasize location and travel (e.g., Google maps [http://maps.google.com]), while thematic mapping sites emphasize data relationships (e.g., the Centers for Disease Control and Prevention's Behavioral Risk Factor Surveillance System [BRFSS] maps at http://apps.nccd.cdc.gov/gisbrfss).

For the second version of the CHSI, an Internet mapping application was developed to provide access to CHSI data. The mapping of public health and other statistical data provides new opportunities for visualization, exploration, and understanding of the data. Many people have great difficulty fully understanding statistical information (3). Providing audiences with results of analyses by using maps is a useful approach for enhancing understanding of complex data sets. The human brain can perceive complex patterns in data more easily when those data are presented in a graphic (in particular, a map) format, as opposed to tabular displays of numeric values (4). By transforming the data from tabular to mapped, the users' perspectives of the data is changed to that of a synoptic overhead view, in which spatial relationships in the data are made evident. Beyond simply being a graphic presentation of data, maps allow the user to find patterns and relationships among the mapped data (5). This capability facilitates a greater understanding of the data and can prompt questions that lead to further inquiry and exploration of the data. Thus, a geographic component can enhance communication of key features in the CHSI data and potentially increase the size and scope of the CHSI audience.

## CHSI GIS Internet Mapping Site: Design Considerations

Both static and dynamic maps are available through the Internet. Static maps, which are found in map and image libraries (e.g., http://www.loc.gov/rr/geogmap), contain maps available in a format that can be viewed, printed, or downloaded but not edited. The strengths of this approach are that the user only has to be familiar with using the Internet and the images can easily be incorporated into a document. The look of the map, its geographic extent, and the presentation of data are fixed and usually of high quality. However, this strength also points to its weakness. All cartographic and data decisions have been made by someone other than the user and thus may not meet the user's exact needs. In contrast, an interactive mapping site permits access to the data and tools needed for manipulating data. The software or the site developer may set some restrictions on the access to the data and the type of products or reports that can be generated. The CHSI GIS Web site is an example of an interactive mapping site that includes the capability to print the maps that are generated by the users.

The development of an Internet map product (i.e., a map library consisting of static maps or an interactive mapping site) begins with the intended audience and how they will use the product. A needs assessment would have been useful for guiding the development of the CHSI GIS Web site but was not feasible at the time of development. As is discussed later, evaluation will be critical for understanding how the site is being used and can be improved in the future. For the initial CHSI GIS mapping site, we focused our efforts on designing a site that would be useful to the primary users of the first version of CHSI, namely, local community groups and local public health staff. We anticipated that the site and CHSI map products would be used in a variety of ways, including printed or electronic maps in internal and external reports and presentations. It is also likely that users will want to conduct exploratory spatial data analyses as a better way to understand the data.

Cartographic cognition is the process by which the human brain recognizes spatial patterns and relationships; this is called geovisualization when using GIS. The geovisualization literature indicates that several factors must be considered in designing map products (5-8). One consideration is color. For example, if using choropleth, (i.e., shaded) maps, the choice of color to display variations in the data is dependent on the media on which the maps are displayed (e.g., computer monitor or paper). For example, the number of colors that can be used in computer displays and in print is governed by the computer's screen and by the number of colors that a printer or plotter is designed to produce. An additional consideration is the color schemes used to represent ordered data (e.g., high to low, more to less). The color sequence should still clearly show the data in the shades of gray produced when the map is printed in black and white. Generally, darker shades are used to represent higher data values and lighter shades to represent lower values. Many cartographers suggest using 4 to 6 classes in a choropleth map (5,9,10). Too few classes might mask any spatial patterns, while too many classes can overwhelm the reader with information or make it difficult to distinguish between the colors (11). Another important consideration in color selection is that approximately 8% of men and 0.5% of women are color-blind, primarily red-green colorblindness (6). Therefore, potentially 1 in 12 people visiting a Web site might be color-blind (4).

Map scale is also a consideration. Although less critical to the CHSI GIS Internet mapping site since the data are county and state level, it is still important for presentation. For example, one of the defining and unique characteristics of CHSI is the concept of peer counties (www.communityhealth.hhs.gov). Peer counties, similar in population composition and selected demographics, are grouped into 88 peer groupings, or strata. Although some peer counties might be in close proximity, all are not likely to be near one another. For example, a number of Strata 1 counties, grouped together because of their large population size, are in southern California (e.g., Orange, San Diego, Los Angeles). Other Strata 1 counties include Cook County, Illinois; Palm Beach County, Florida; King County, Washington; and New York County, New York. A small-scale map (Figure) adequately captures the location of peer counties in a stratum but does not provide visual insight into how the counties compare to one another. A peer-county map also does not show how one county compares to its neighboring counties. Past users of CHSI said that this comparison was of interest to them.

**Figure. Community Health Status Indicators (CHSI) — Strata 1, Peer Countiesa:**
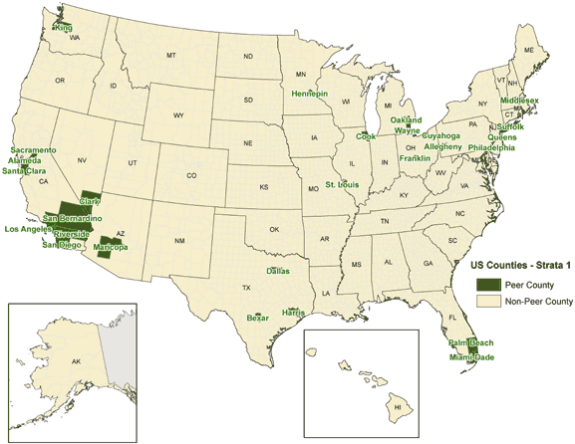
^a^Peer counties are similar in population composition and selected demographics. There are 88 peer groupings, or strata. Strata 1 counties are grouped together because of large population size. Since CHSI was developed in 2000, some of the 34 counties in Strata 1 have been surpassed in population by counties that were not originally included in that peer grouping.

### CHSI GIS Analyst: Version 1

#### Content

The CHSI GIS Analyst application was conceived of as an easy-to-use Web-based GIS application that would accompany the CHSI report and increase accessibility to the information held in the report through the coordinated use of both map and tabular displays. The guiding premise for the development of the CHSI GIS Analyst was that the site must be simple to use. The positive characteristics of the first version of the CHSI hard-copy county report were its simplicity, ease of use, and organization. Thus, the site designers wished to mirror these characteristics within the design of the Web-based GIS application.

Each indicator in the CHSI report can be mapped and compared visually to other areas, including peer counties and neighboring counties. The application is driven by the selection of indicators from 1 of the 9 indicator groups (Demographics, Summary Measures of Health, National Leading Causes of Death, Measures of Birth and Death, Vulnerable Populations, Environmental Health, Preventive Services Use, Risk Factors for Premature Death, and Access to Care). For most of the CHSI data, the indicator is displayed as a choropleth map classified according to its percentile rank out of all U.S. counties into 1 of 4 categories for the variable: counties in the 10th percentile, counties between the 10th and 50th percentile, counties from the 50th to the 90th percentile, and counties in the 90th percentile. Fixing the data categories to percentile ranks gives the user an easily interpreted indicator, by showing the position of the county above or below the median for all U.S. counties and whether it is at the top or bottom 10%. A few of the indicators are not continuous (e.g., some of the environmental health indicators are measured as dichotomous variables) or are measured only at the state level (e.g., data on the percentage of smokers from BRFSS). In these cases, the indicators are not categorized by percentile rank but by actual value.

The CHSI GIS Analyst Web site can be opened from a link on the CHSI report Web site (www.communityhealth.hhs.gov). Three tabbed interfaces are available to the user: Indicator View, Peer County View, and State View.

In the Indicator View tab, up to 4 indicators can be selected and displayed separately for an index county. The application default is to map the indicator and its neighboring counties in each of 4 map boxes. An exception to this is the state-level CHSI. In that case, the default is the state and its contiguous states. Each of the map boxes in the mapping display panel can use zoom and pan tools to navigate to the user's area of interest.

Selection of the Peer County View tab enables additional mapping and graphing capabilities. This section focuses on the relationships between the peer counties. For example, a map and listing of all peer counties can be generated in one frame and a choropleth map of a peer county displaying a selected indicator in another frame. That second frame can be switched to a graphic of the range of values for peer counties in that stratum. Thus, the user is getting information on percentile rank of the county and its actual value.

Finally, the State View tab is similar to the Peer County View, but the focus is on the index county and its spatial relationship to all other counties within the state. The choropleth mapping still represents the percentile rank of the county out of all U.S. counties but the graphic of the range of values is specific to counties within a state.

#### Technical Specifications

The CHSI GIS Analyst, which is simply a Web-based GIS application, employs a commonly used application model, the three-tier model. This model emphasizes the division of an application or system into three "tiered" layers: a data tier, a business tier, and a presentation tier (12). The data tier is composed of the data storage components employed to store the application data. The business tier is composed of the business logic that is employed to access the data, manage the data, and package the data for use and presentation in the presentation tier. The presentation tier is composed of the actual view, or "graphical user interface" that the user sees and manipulates. This tier is responsible for drawing the user interface and accepting user requests, often by a host of controls such as buttons, drop-down lists, and context-sensitive menus. The three-tier model has been accepted and extensively used within the information technology community because it allows for any tier to be upgraded or replaced independently without significant disruption in the functioning of the entire system or application. Thus, the three-tier approach facilitates the complete replacement of the application interface with a new interface that includes alternate or improved methods of data visualization. Additionally, the tiered approach can facilitate the expansion of data (or upgrade to a new version of data) without compromising the integrity of the application and presentation tiers that depend on that data.

The data tier used by CHSI GIS Analyst includes a Microsoft SQL Server 2003 relational database. This relational database contains the tabular as well as the geospatial data that form the foundation of the application. The development team used Environmental Systems Research Institute, Inc. (ESRI) Spatial Database Engine 9.1 (SDE) to store geospatial features within the structure of the Microsoft SQL Server database. The SDE software facilitates the storage of geospatial data within the SQL Server relational database, and it stores the data in such a way that the entire suite of ESRI GIS products can access and map the data.

The business tier includes Microsoft .NET 2003, ESRI's ArcIMS 9.1, and an ArcIMS Connector, which is a custom-built component set that facilitates the communication from Microsoft .NET components to the ArcIMS server. The components in the business tier work together to process requests, pull the appropriate data that is required for maps and reports, and generate map images that are subsequently integrated into the graphical user interface.

The presentation tier includes Internet browsers that are on the market today such as Microsoft's Internet Explorer, Netscape's Navigator, and Mozilla's Firefox. This tier receives information in the form of HTML and images from the business tier. It is responsible for rendering the interface as prescribed by the business tier, accepting user interactions, and communicating user requests back to the business tier.

A tabbed interface approach was used for two reasons. First, the tabbed interface enables the addition of map/report displays easily and simply; another tab need only be added to provide new reporting functionality. Second, users interact with tabs in a multitude of different desktop and Web-based applications and are familiar with the tabbed interface concept and its practical usage (13).

### Summary and Discussion

The second version of the CHSI, which includes an Internet mapping application, has taken advantage of the more widespread familiarity and use of GIS technology within the public health community. The CHSI GIS Analyst application was planned by the workgroup to highlight spatial relationships between peer and contiguous counties, promote spatial data exploration, produce maps and graphs of sufficient quality to be included in presentations and reports, and be simple to navigate. Although the workgroup has extensive experience in public health data and GIS applications, to adequately gauge our success in these efforts requires input from the community of CHSI GIS users. As noted previously, because of a lack of time and resources, a needs assessment was not feasible for the initial version of the CHSI GIS Analyst. To fill this gap, we propose some next steps for soliciting user input that will likely enhance and sustain the CHSI GIS Analyst application.

In addition to our own experiences, we have benefited from others' cognitive and cartographic research in planning and designing the CHSI GIS Web site (1,5,6). This information has provided a solid evidence-based foundation to build an overarching framework for the CHSI GIS Analyst. However, we also suggest that efforts and resources be directed toward data collection to answer questions specific to the CHSI GIS Analyst including who is using the GIS Web site, how they are using the site, how does the mapping application add or compare to the report itself, is the site easy to navigate and understand, and what do they like or not like about the site. Limited information can be obtained from Web site statistics (e.g., the number of hits to the Web site) and by including a link for users to provide feedback. However, systematic data collection, through the use of focus groups and user surveys, is needed. This information will provide important insight into how effective we are in conveying the indicators to the intended audience. Although critical for CHSI, it also has implications beyond this project as more public health agencies present their data in map format. If we know more about how people use, respond to, and interpret maps, especially thematic maps, then we are in a better position to communicate public health data for policy and action.

## References

[1] Pickle LW, Mungiole M, Jones GK, White AA (1996). Atlas of United States mortality.

[2] Devesa SS, Grauman DJ, Blot WJ, Pennello G, Hoover RN, Fraumeni JF (1999). Atlas of cancer mortality in the United States, 1950–1994.

[3] Bell SB, Hoskins RE, Pickle LW, Wartenberg D (2006). Current practices in spatial analysis of cancer data: mapping health statistics to inform policy makers and the public. Int J Health Geogr.

[4] MacEachren AM (1995). How maps work: representation, visualization, and design.

[5] Dent BD (1999). Cartography: thematic map design.

[6] Brewer CA (2005). Designing better maps.

[7] MacEachren AM, Buttenfield BP, Campbell JB, DiBiase DW, Monmonier M, Abler RF, Marcus MG, Olson JM (1992). Visualization. Geography's inner worlds: pervasive themes in contemporary American geography.

[8] Slocum TA, McMaster RB, Kessler FC, Howard HH (2005). Thematic cartography and geographic visualization.

[9] Monmonier MS (1977). Maps, distortion, and meaning: resource paper No. 75-4.

[10] Gilmartin P, Shelton E (1989). Choropleth maps on high resolution CRTs: the effects of number of classes and hue on communication. Cartographica.

[11] ColorBrewer.

[12] Meijer E, van Velzen D (2001). Haskell server pages: functional programming and the battle for the middle tier. Electronic Notes in Theoretical Computer Science.

[13] Spolsky J (2001). User interface design for programmers.

